# Research progress on the mechanism and markers of metabolic disorders in the occurrence and development of cognitive dysfunction after ischemic stroke

**DOI:** 10.3389/fendo.2025.1500650

**Published:** 2025-01-22

**Authors:** Huaqiang Li, Xiaohua Ke, Bianying Feng, Huan Tian, Zhenzhen Cai, Anren Zhang, Qiuhong Man

**Affiliations:** ^1^ Department of Rehabilitation Medicine, Shanghai Fourth People’s Hospital, School of Medicine, Tongji University, Shanghai, China; ^2^ School of Health Preservation and Rehabilitation, Chengdu University of Traditional Chinese Medicine, Chengdu, China; ^3^ Department of Clinical Laboratory, Shanghai Fourth People’s Hospital, School of Medicine, Tongji University, Shanghai, China

**Keywords:** ischemic stroke, cognitive dysfunction, metabolic disorders, biomarkers, review

## Abstract

Post-stroke cognitive impairment (PSCI) is a common complication following a stroke that significantly affects patients’ quality of life and rehabilitation outcomes. It also imposes a heavy economic burden. There is an urgent need to better understand the pathophysiology and pathogenesis of PSCI, as well as to identify markers that can predict PSCI early in the clinical stage, facilitating early prevention, monitoring, and treatment. Although the mechanisms underlying PSCI are complex and multifaceted, involving factors such as atherosclerosis and neuroinflammation, metabolic disorders also play a critical role. This article primarily reviews the relationship between metabolic disorders of the three major nutrients—sugar, fat, and protein—and the development of cognitive dysfunction following ischemic stroke (IS). It aims to elucidate how these metabolic disturbances contribute to cognitive dysfunction post-stroke and to explore potential metabolic biomarkers for PSCI. We believe that this review will offer new insights into the early identification, treatment, and prognostic assessment of PSCI.

## 1 Introduction

Ischemic stroke (IS) refers to the ischemic necrosis of brain tissue caused by thrombosis or embolic occlusion of cerebral blood vessels, accounting for 62.4% of all stroke cases (1). In recent years, the incidence of IS has been rising among young and middle-aged individuals (2) Furthermore, the risk of stroke increases progressively with age. In the future, the combination of population growth and aging may result in a significant rise in both mortality and disability rates among IS patients worldwide.

Stroke patients often experience multiple functional deficits as a result of acute clinical events, with approximately one-third of them developing post-stroke cognitive impairment (PSCI) (3). PSCI leads to increased disability, malnutrition, and a greater family and social burden (4, 5). However, there is currently no specific medication for the treatment of PSCI, and interventions primarily focus on preventing and slowing cognitive decline. Additionally, the pathogenic mechanisms and biomarkers for predicting the progression of PSCI remain unclear, presenting significant challenges for clinical prevention, diagnosis, and treatment.

Metabolism refers to a series of ordered chemical reactions that occur in organisms to maintain life. It is the process by which organisms continuously exchange matter and energy. When this exchange is disrupted, it can lead to the onset and progression of various diseases (6, 7). Many phenotypes in humans and other organisms involve changes in metabolism, and metabolites found in body fluids can reflect the connections between genotype, environment, and phenotype in different disease states. Therefore, metabolic markers can provide unique insights into the mechanisms of disease development, making them valuable biomarkers for disease diagnosis, prediction, and treatment research (8–11). As is well known, sugar, fat, and protein are the three major energy sources in the human body (7). Among them, sugar is the primary energy source, fat serves as an alternate energy source, and protein is primarily used for constructing and repairing body tissues. These energy substances work in concert within the body to maintain normal physiological functions, and metabolic disorders involving these three substances are closely associated with a variety of diseases, including cardiovascular disease and diabetes (12–14). Similarly, metabolic disorders are also observed in stroke patients with cognitive impairments.

Based on the above understanding, this review aims to comprehensively and systematically elucidate the association and mechanisms between metabolic changes in sugar, fat, and protein and the progression of cognitive dysfunction in patients with PSCI following IS. It will analyze the relationships and interactions among related metabolic biomarkers, to provide new perspectives for the early diagnosis and treatment of PSCI.

## 2 Glucose metabolism and PSCI

### 2.1 Role of disorders of glucose metabolism in cognitive dysfunction after IS

Glucose is the primary form of carbohydrate utilized by the human body after metabolic conversion, and it is primarily metabolized through aerobic oxidation, glycolysis, and the pentose phosphate pathway (15). In the brain, glucose and oxygen levels are crucial for the growth and function of neurons (16, 17). When IS occurs, cerebral blood flow is interrupted, blocking the transportation of oxygen and nutrients (such as glucose and vitamins). As a result, glucose metabolism shifts from aerobic to anaerobic pathways. This shift reduces ATP production, increases lactate production, inhibits the tricarboxylic acid cycle, and disrupts cerebral homeostasis (18, 19). Both glucose and lactate serve as energy substrates for various types of memory neurons (20), with lactate being essential for cognitive tasks that require high attention and corresponding hippocampal enhancement *in vivo*. Anaerobic glycolysis can benefit cognitive ability by supporting energy production (20). However, during IS, reduced cerebral perfusion and impaired glucose transport (21), lead to the inhibition of both aerobic and anaerobic glycolysis, which in turn impairs the energy supply to memory neurons and contributes to cognitive dysfunction.

The pentose phosphate pathway (PPP) is a branch of glucose metabolism that produces ribose 5-phosphate (R-5-P) and nicotinamide adenine dinucleotide phosphate (NADPH) (22). During the acute phase of cerebral ischemia-reperfusion, glucose 6-phosphate dehydrogenase (G6PD), a key enzyme in the PPP pathway, can be activated by ataxia telangiectasia mutated (ATM) kinase. This activation occurs through the phosphorylation of serine 85 (S85) on heat shock protein 27 (HSP27), which in turn initiates the body’s antioxidant protection mechanisms (23). Therefore, the PPP pathway is activated following cerebral ischemia (19). R-5-P and NADPH are essential for the synthesis of nucleic acids in the body and play significant roles in protein synthesis and translation. Memory is closely linked to both protein synthesis and degradation, and cognitive dysfunction can occur when these processes are dysregulated (24) Consequently, patients with PSCI may experience disorders in the PPP pathway, leading to an imbalance between protein synthesis and degradation (25), resulting in cognitive dysfunction.

Glycogen is a branched-chain polymer of glucose that plays a major role in energy storage in eukaryotes. Glycogen synthase (GS) is a key enzyme in glycogen synthesis, while glycogen phosphorylase (GP) is a key enzyme in glycogenolysis (26, 27). GS activity is regulated by the dephosphorylation of glucose 6-phosphate (G6P) and the inhibition of its phosphorylation. In the brain, GS is activated in astrocytes following IS through a cascade involving protein kinase A (PKA), glycogen phosphorylase kinase (PhK), and glycogen phosphorylase (GP). The activation of glycogen synthase kinase-3β (GSK3β) also contributes to the accumulation of glycogen by inactivating PhK and GP (28). This accumulation of glycogen inhibits glycogen breakdown and the supply of energy to the brain during acute cerebral ischemia, which can lead to cognitive impairment.

**Figure 1:**
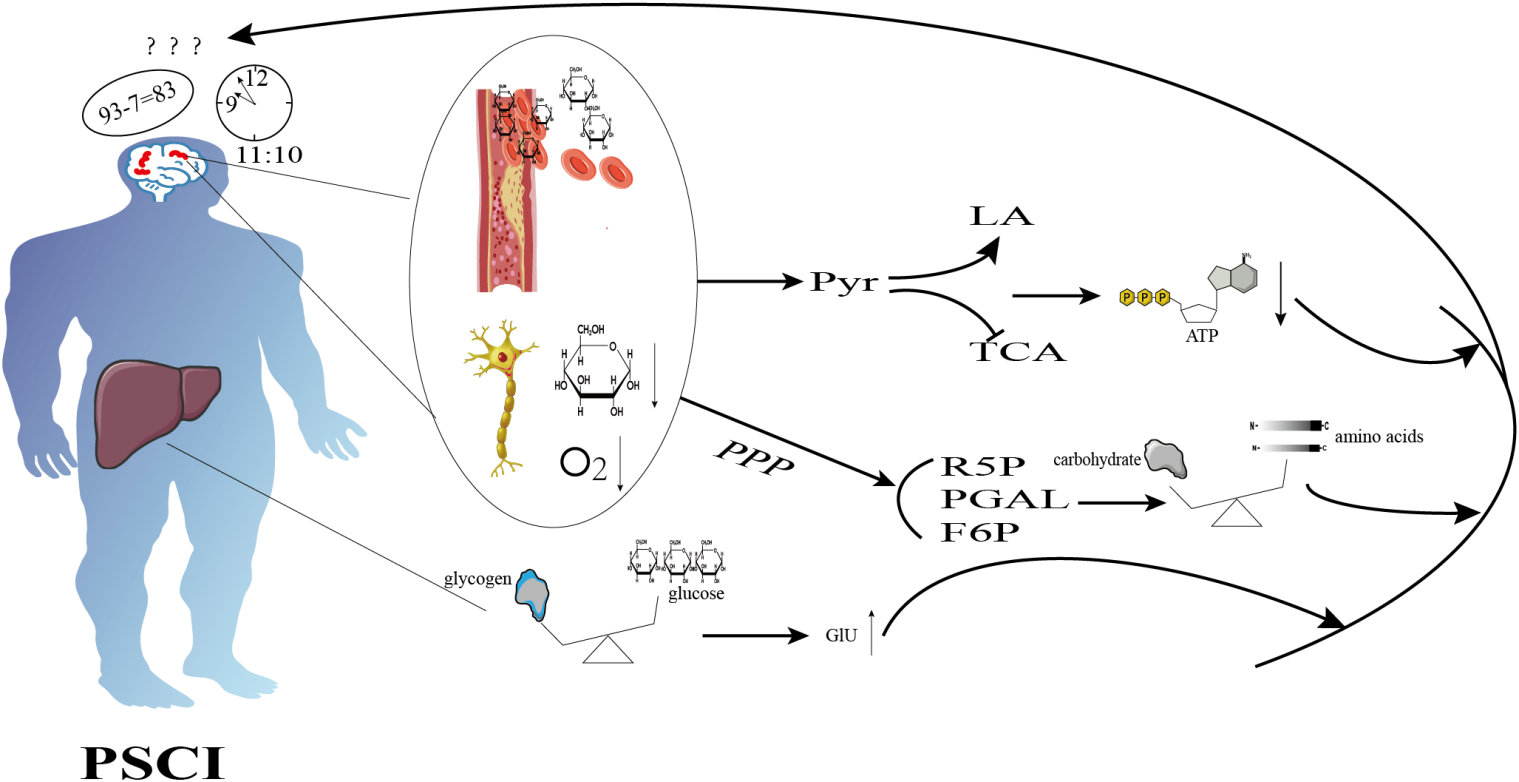
Schematic diagram illustrating the mechanisms of glucose metabolism in cognition dysfunction after ischemic stroke. Ischemic stroke inhibits aerobic glycolysis, promotes anaerobic glycolysis, and reduces ATP production. It also enhances the pentose phosphate pathway, resulting in an imbalance between protein synthesis and degradation, as well as disturbances in glycogen metabolism, which ultimately leads to hyperglycemia. These alterations contribute to cognitive dysfunction.

Glycolysis mainly refers to the metabolic process of synthesizing glucose from non-sugar substances such as amino acids, pyruvate, and glycerol in the body. Glucose-6-phosphatase, fructose diphosphatase-1, pyruvate carboxylase, and phosphoenolpyruvate carboxylase are the key enzymes in the process of gluconeogenesis, and gluconeogenesis may be altered when the activity of any of them is affected (29). Animal studies found that during the acute phase of IS, rats had elevated fasting glucose and insulin levels and up-regulated expression of hepatic gluconeogenesis genes, including phosphoenolpyruvate carboxykinase, glucose-6-phosphatase, and fructose-1,6-bisphosphatase, and hepatic gluconeogenesis-associated positive regulators, which suggests that IS may promote gluconeogenesis through activation of the key gluconeogenic enzymes and regulators, and make up *in vivo* for the organism’s missing energy for anaerobic glycolytic metabolism (30). The body’s physiological concentration of blood glucose fluctuates within a certain range and is regulated by glycogen synthesis and catabolism, and it has been found that patients with IS tend to be hyperglycemic (31), and they may have disorders of glucose metabolism. Hyperglycemia may lead to insulin resistance (32) and atherosclerosis (33), both of which are closely related to cognitive impairment (34, 35). Specific metabolic changes are shown in **Figure 1**.

### 2.2 Glycometabolic markers associated with PSCI

Blood glucose is the amount of glucose in the blood, and it is an important indicator for assessing the normalcy of glucose metabolism (36). Hyperglycemia has been found to occur after IS in most patients in the absence of diabetes (37, 38), which is an independent risk factor for cognitive dysfunction 6 months after IS and can be used as a predictor of PSCI at 3 months (39, 40). The body experiences stress-responsive elevation of glucose after IS, and in the state of ischemia and hypoxia, the anaerobic fermentation of glucose is accelerated, and the level of lactic acid rises markedly, causing acidosis. Acidosis further promotes biochemical changes in the ischemic area, exacerbates cerebral tissue hypoxia and edema, and leads to enlargement of the infarct foci (41). In addition, hyperglycemia attenuates the therapeutic effect of early tissue plasminogen activator (IV-tPA) in patients with IS and contributes to the alteration of blood-brain screen permeability in the context of tPA (42, 43), both of which cause cognitive dysfunction.

Glycosylated hemoglobin (HbA1c) is formed when hemoglobin in red blood cells combines with sugars, primarily glucose, in serum through a non-enzymatic reaction (44). A nonlinear relationship between HbA1c and PSCI was found to exist 3-6 months after IS, with a positive correlation between HbA1c and PSCI when HbA1c was greater than 8.2% and a 0.96-fold increase in the risk of PSCI for every 1% increase in HbA1c (45). A cohort study found that HbA1c was an independent risk factor for cognitive impairment 6-12 months after acute mild IS (46). Xu et al. suggested that cognitive impairment was associated with insulin dysregulation and increased expression of insulin-degrading enzymes (45). Gong et al. found that serum HbA1c had a sensitivity and specificity of 67.9% and 82.8%, respectively, with an AUC of 0.829 for predicting the development of PSCI 6-12 months after stroke (46), and the relationship between glycated hemoglobin and PSCI should be further explored in the future.

## 3 Lipid metabolism and PSCI

### 3.1 Mechanism of lipid metabolism disorders in cognitive dysfunction after IS

Lipid diets are an essential source of energy and nutrition in human life, and they play an important role in the prevention or treatment of chronic diseases (47). Some studies have reported that saturated fatty acids help improve memory and exogenous fatty acids slow cognitive decline (48, 49). Currently, omega-3 and omega-6 fatty acids are used in the clinical treatment of patients with cognitive impairment (50). The metabolism of lipids in the human body is mainly a process of fatty acid synthesis and fatty acid β-oxidation (51). In organisms, acetyl coenzyme A, ATP, and NADPH are involved in fatty acid synthesis as raw materials, in which acetyl coenzyme A needs to be transferred from mitochondria to the periplasm to generate fatty acids through the citric pyruvate cycle, and acetyl coenzyme A carboxylase is the key enzyme in this synthesis pathway, and the enzyme loses its activity after phosphorylation and glucagon and epinephrine can make the enzyme phosphorylated to be ineffective, while insulin can Insulin enhances the enzyme activity to promote fatty acid synthesis. After IS, due to hypoxia, the glucose metabolic pathway is shifted to anaerobic glycolysis, ATP and acetyl coenzyme A production is reduced, and the tricarboxylic acid cycle is inhibited (30), which may hinder fatty acid synthesis by reducing the raw materials for fatty acid synthesis. In addition, it has been found that acute events of stroke cause activation of the hypothalamic-pituitary-adrenal (HPA) axis (52), the elevation of cortisol and glucagon, which in turn may prevent fatty acid synthesis by phosphorylating acetyl coenzyme A carboxylase. In summary, fatty acid synthesis may be inhibited after the onset of IS.

Fatty acid β-oxidation (FAO) consists of three major steps: activation of fatty acids, transfer of lipoyl CoA, and β-oxidation of lipoyl CoA. The activation phase is catalyzed by lipoyl CoA synthetase located in the endoplasmic reticulum and the outer mitochondrial membrane in the presence of ATP, CoA-SH, and Mg^2+^ to generate lipoyl CoA, and the activated lipoyl CoA has to be transferred to the mitochondria with the help of carnitine-lipoyltransferase 1 (the key enzyme). After entering the mitochondrial matrix, lipoyl CoA undergoes a biochemical reaction catalyzed by the fatty acid β-oxidation enzyme system while releasing a large amount of energy, and carnitine lipoyltransferase 1 is the key enzyme in the process of β-oxidation (53). In the vigorous glucose metabolism, acetyl CoA, citrate increase can allosterically activate acetyl CoA carboxylase, promote malonate monoacyl CoA synthesis, and competitively inhibit carnitine lipoyltransferase 1. Post-stroke inhibition of aerobic oxidation, weakening of glucose metabolism, inhibition of the tricarboxylic acid cycle, and reduction of the production of acetyl CoA, citrate, etc. (30) may lead to a reduction in malonic acid monoacyl CoA synthesis, and a decrease of competitive inhibition, and therefore may enhance fatty acid beta-oxidation. Thus it may lead to enhanced fatty acid β-oxidation.

**Figure 2:**
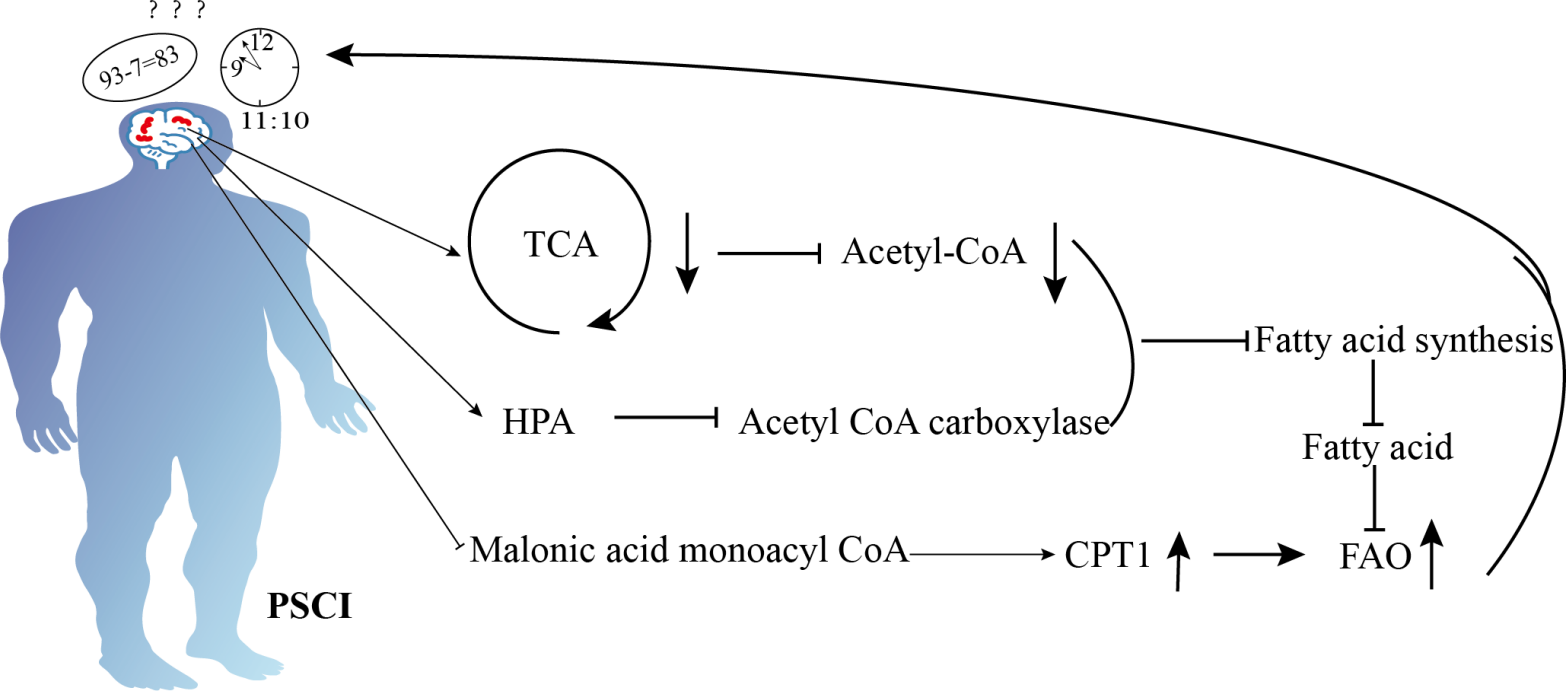
Schematic diagram illustrating the mechanisms of lipid metabolism in cognition dysfunction after ischemic stroke. Ischemic stroke enhances fatty acid β-oxidation to produce energy by inhibiting malonyl-CoA, but ischemic stroke also suppresses the tricarboxylic acid cycle, reduces acetyl-CoA, activates the hypothalamic-pituitary-adrenal axis, and inhibits acetyl-CoA carboxylase. These mechanisms decrease fatty acid synthesis, reducing the availability of fatty acids and ultimately leading to impaired energy supply, which results in cognitive dysfunction.

Animal studies have shown that reduced peroxisomal β-oxidation enhances amyloid-β production and leads to spatial memory deficits in diabetic rats through oxidative stress (54). Peroxisome proliferator-activated receptor α (PPARα) is a key transcription factor involved in fatty acid oxidation. It facilitates the transfer of fatty acids into mitochondria for oxidation. In postoperative cognitive dysfunction (POCD) mice, pretreatment with the PPARα agonist FF prevents long-term isoflurane anesthesia-induced POCD by enhancing fatty acid oxidation (55). Similarly, it was found that SUL-138 increased the levels of proteins involved in fatty acid degradation and oxidation, improving cognitive function in both wild-type and APP/PS1 mice (56). Furthermore, fatty acid β-oxidation generates a significant amount of energy for the organism, suggesting that it may contribute to the protection of cognitive function after IS. However, due to the inhibition of fatty acid synthesis following stroke, the corresponding β-oxidation may also be suppressed, thereby losing its protective effect and potentially leading to cognitive dysfunction. Specific metabolic changes are depicted in **Figure 2**.

### 3.2 Lipid metabolism biomarkers associated with PSCI

Triglycerides (TGs) are an efficient source of energy in the body (57). Both fasting triglycerides (FTGs) and non-fasting triglycerides (NFTGs) levels were found to be elevated after IS (58). Triglycerides may promote amyloid-β (Aβ) protein deposition by affecting membrane fluidity and by acting as a carrier for Aβ-protein aggregation, thereby influencing Aβ aggregation kinetics (59). Aβ-protein deposition has also been observed in the brains of IS patients during autopsy (60). Aβ-protein can disrupt cerebrovascular homeostasis by directly interacting with blood-brain barrier (BBB) cellular components or by promoting neuroinflammation, which increases BBB permeability (61, 62). Studies have shown that increased BBB permeability can lead to cognitive dysfunction (63).

Triglycerides (TGs) circulate in the blood as triglyceride-rich lipoproteins (TRL). When circulating TG levels are elevated, TRL and their residual particles can accumulate in the plasma, penetrate the intimal layer of the artery, and, once embedded in the subendothelium, TRL can be scavenged by resident macrophages. This contributes to the formation of foam cells and the progression of atherosclerotic disease (64, 65). Additionally, TRL remnants, although larger than low-density lipoproteins (LDL), are smaller than their natural counterparts—very low-density lipoproteins (VLDL) and chylomicrons. These remnants are relatively cholesterol-rich and can enter the arterial wall, where they are ingested by macrophages, leading to cholesterol accumulation in atherosclerotic lesions (57, 66). Atherosclerosis may cause cognitive impairment through chronic cerebral underperfusion and cerebral embolism (35). Cheng et al. found that a high triglyceride-to-glucose ratio on admission was associated with cognitive impairment and was able to predict the occurrence of PSCI after 3 months (67, 68). In a Meta-analysis study by Kim et al., triglycerides were also recommended as a potential metabolic marker for PSCI (69). Feng et al. used recombinant human growth hormone to treat patients with PSCI, which significantly reduced triglyceride levels and improved Moca scores in patients (70). Future studies should be conducted on triglycerides to explore their up-to-value as a biomarker for PSCI.

Low-density lipoprotein (LDL) is the end product of lipoprotein degradation and is closely associated with cardiovascular disease (71). Numerous studies have found that high LDL levels cause cognitive dysfunction (72, 73), and LDL levels tend to be elevated in patients with IS (74). Jurcau et al. reported that LDL is associated with PSCI (75), but other studies have found no correlation between LDL and cognitive decline in stroke (76, 77). The relationship between LDL and cognitive impairment remains inconclusive and requires further investigation. Atherosclerosis (AS), a high-risk factor for IS, involves LDL in its atherogenic processes. While natural LDL does not exert atherogenic mechanisms *in vitro*, it is modified to oxidized LDL (Ox-LDL) under oxidative stress. Ox-LDL can promote atherosclerosis by reducing nitric oxide (NO) production and antioxidant enzymes, which impair endothelial integrity (78). Furthermore, atherosclerosis itself can contribute to cognitive dysfunction (35). Feng et al. treated PSCI patients with recombinant human growth hormone, which significantly reduced LDL levels and improved MOCA scores (70).

Non-HDL cholesterol (non-HDL-C) refers to the sum of cholesterol contained in lipoproteins other than HDL, primarily including low-density lipoprotein cholesterol (LDL-C) and very low-density (79). lipoprotein cholesterol (VLDL-C) levels of non-HDL-C lipoproteins in IS are associated with more severe cognitive dysfunction, showing a positive correlation between the two (80). Similarly. reported that elevated serum non-HDL-C levels significantly increase the risk of cognitive dysfunction following acute ischemic stroke (AIS). They suggested that serum non-HDL-C includes all known or potentially atherosclerotic fatty particles, which may promote atherosclerosis (81). In a study by Jiaolving 583 AIS patients diagnosed with post-stroke cognitive impairment (PSCI), it was found that using 3.52 mmol/L as the cutoff for serum non-HDL-C levels, the sensitivity and specificity for diagnosing PSCI were 90.3% and 63.7%, respectively, with an area under the curve (AUC) of 0.773 (81).

## 4 Protein metabolism and PSCI

### 4.1 Mechanisms of protein metabolism disorders in cognitive dysfunction after IS

Proteins are integral components of the organism, providing essential nutrients and maintaining protein homeostasis primarily through degradation and synthesis pathways, which help keep the body healthy (82, 83). The endoplasmic reticulum is a eukaryotic organelle within the cytoplasm that produces and modifies proteins. When IS occurs, the delivery of oxygen and glucose to neural tissues can be stopped to trigger the endoplasmic reticulum stress cascade, which rapidly induces protein misfolding and endoplasmic reticulum stress, and when blood flow is restored, reperfusion of the affected tissues leads to oxidative stress as well as alterations in the redox state of the endoplasmic reticulum, which disrupts protein disulfide bonding, leading to misfolding of endoplasmic reticulum proteins (84, 85). To mitigate this damage, eukaryotic cells activate the unfolded protein response (UPR). The UPR helps alleviate ER stress by upregulating cofactors, chaperones, and enzymes in the ER, while simultaneously downregulating protein translation. This response provides neuroprotection during IS by addressing the protein folding defects induced by the stressor. However, prolonged or excessive ER stress can overwhelm the UPR, rendering it ineffective (30, 86).

Furthermore, the activation of the pentose phosphate pathway (PPP) due to impaired oxygen transport after IS (19) leads to the production of ribose 5-phosphate (R-5-P) and nicotinamide adenine dinucleotide phosphate (NADPH). Consequently, both ER stress and PPP activation after IS may result in disturbances in protein synthesis and degradation, which can contribute to cognitive dysfunction (87).

Protein synthesis and degradation are both ATP-consuming processes and are therefore closely regulated by energy metabolism. Following IS, ATP production is reduced due to changes in glucose metabolic pathways (18), which in turn can regulate both protein synthesis and degradation. Additionally, the endoplasmic reticulum (ER) undergoes stress after IS (88), and the eukaryotic translation initiation factor 2α (eIF2α) plays a role in the morphology and function of vascular smooth muscle cells in atherosclerotic plaques under the influence of ER stress. Elevated expression of eIF2α has been shown to alleviate the progression of atherosclerosis (89). However, patients with IS often exhibit severe atherosclerosis, which may result in decreased levels of eIF2α. Furthermore, stress-induced phosphorylation of eIF2α further inhibits protein translation and reduces protein synthesis.

TORC1 is a key regulator of cell growth, controlling processes such as protein biosynthesis, transcription, nutrient uptake, energy expenditure, and autophagy activity. Two central downstream effector proteins of TORC1, the eukaryotic translation initiation factor 4E-binding protein (4E-BP) and ribosomal protein S6 kinase 1 (S6K1) regulate protein biosynthesis and promote its synthesis when activated (90). In contrast, mTORC1 activity has been found to be reduced after IS (91), which leads to the inhibition of protein synthesis.

**Figure 3:**
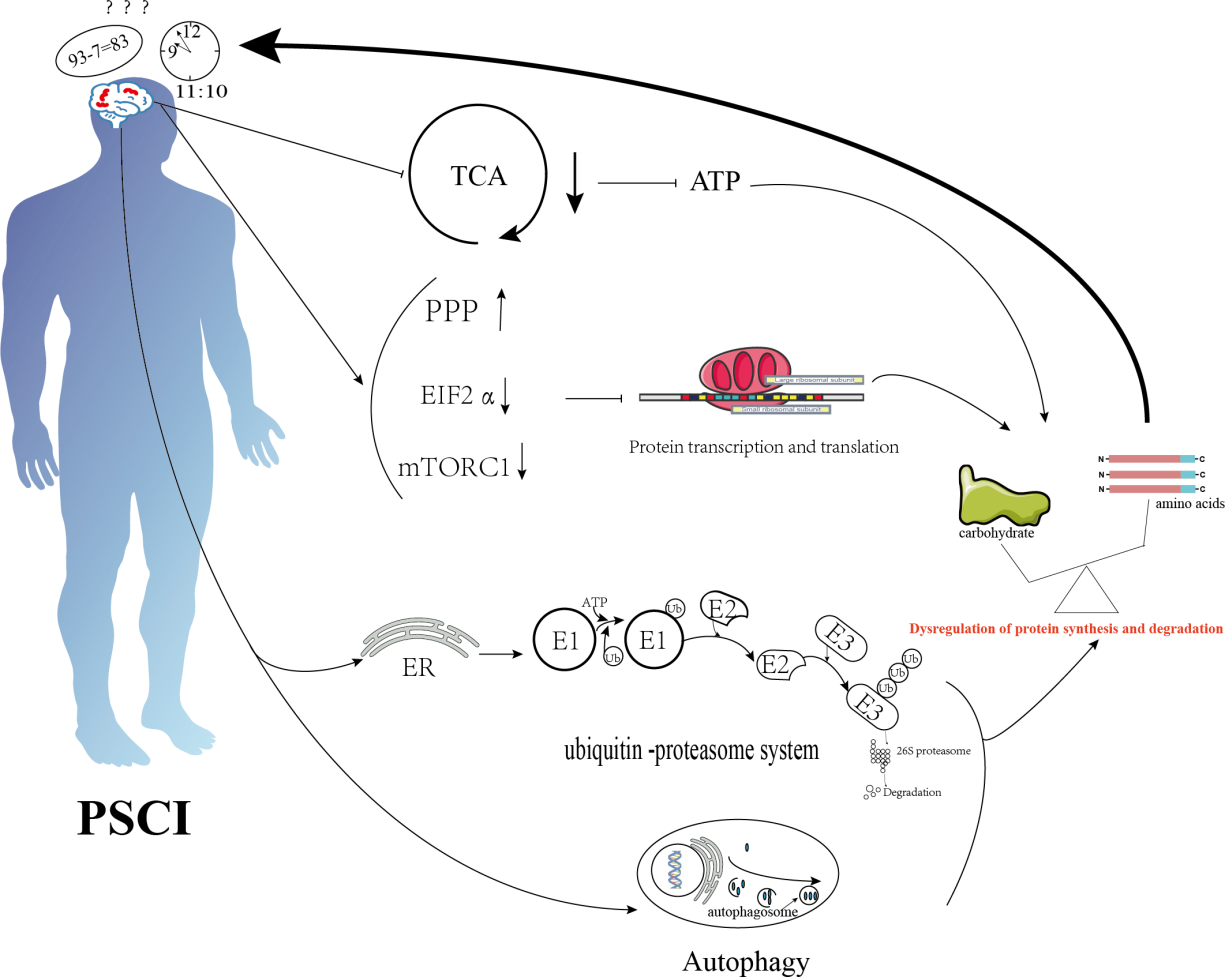
Schematic diagram illustrating the mechanisms of protein metabolism in cognition dysfunction after ischemic stroke. Ischemic stroke suppresses the tricarboxylic acid cycle, activates the pentose phosphate pathway, and inhibits transcription initiation factor 2α and the mammalian target of rapamycin complex 1 (mTORC1), thereby inhibiting protein synthesis. It also promotes protein degradation through endoplasmic reticulum stress and autophagy. The combined effects of these mechanisms lead to an imbalance between protein degradation and synthesis, resulting in cognitive dysfunction.

The ubiquitin-proteasome system (UPS) and autophagy are two major protein degradation pathways in eukaryotic cells (92). The emergence of endoplasmic reticulum stress (ERS) after stroke further activates the unfolded protein response (UPR), which is associated with reduced translational activity, increased protein folding capacity, and the activation of protein degradation pathways (88). Additionally, autophagy is activated after stroke, further promoting proteolysis (93). The process of memory consolidation is closely related to both protein synthesis and degradation (24). In summary, protein synthesis is inhibited and degradation is enhanced after IS, disrupting the balance of protein metabolism and leading to cognitive dysfunction (87). Specific metabolic changes are shown in **Figure 3**.

### 4.2 Protein metabolic markers associated with PSCI

Elevated levels of homocysteine, a non-proteinogenic sulfhydryl-containing amino acid derived from methionine, are known as hyperhomocysteinemia and are closely associated with neurovascular diseases, dementia, and other disorders (94). Increased homocysteine (HCY) levels have been observed in the blood of IS patients (95), and higher HCY levels correlate with more severe cognitive dysfunction, with a positive association between the two (80). HCY has also been recommended as a potential metabolic marker for PSCI in a meta-analysis by Kim et al (69). However, one study found that the relationship between HCY levels and cognitive impairment was age-dependent, being more pronounced in individuals under 65 years of age. This relationship disappeared after adjusting for confounders in the population over 65 years of age (96).

In addition, Li et al. found that homocysteine (Hcy) was associated with PSCI in males, but not in females. This difference may be due to the weakened protective effect of estrogen in females after menopause (97). The presence of Hcy has been shown to exacerbate amyloid-beta (Aβ)-induced apoptosis of brain endothelial cells and promote blood-brain barrier (BBB) disruption in animal studies (98). Furthermore, excessive Tau protein and phosphorylated Tau protein aggregation have been linked to blood-brain barrier disruption (99). Elevated Hcy levels also lead to abnormally reduced vasodilatory responses and compromised cyclooxygenase (CO) synthase activity. This exacerbates endothelial damage, further impairing endothelial function, which facilitates the shedding of large numbers of endothelial cells and promotes the development of atherosclerosis (AS) (80). Therefore, homocysteine may induce cognitive dysfunction by disrupting the blood-brain barrier and promoting the progression of atherosclerosis.

Galactoglucan lectins are a family of carbohydrate-binding proteins found in vertebrates, characterized by significant abundance and diversity in their structure, ligand-binding properties, and physiological functions. Galectin-3, a member of this family, is notably elevated after IS (100). It tends to cause neurotoxic effects during the acute inflammatory phase while exerting neuroprotective effects in the subacute phase by promoting the polarization of classical pro-inflammatory M1 microglia to the anti-inflammatory M2 phenotype, which has healing activity (101). Qian Wang et al. found that the incidence of PSCI increased progressively with the elevation of serum Gal-3 levels (102). Elevated Gal-3 levels are also present in atherosclerotic (AS) vessels, where they affect endothelial cells, macrophages, and vascular smooth muscle cells (VSMCs), all of which are involved in the development of atherosclerosis (103, 104). Atherosclerosis can lead to cognitive dysfunction by restricting blood flow and nutrient supply to the brain. Moreover, Qian Wang et al. found that serum Gal-3 levels could be used to diagnose PSCI, with a cutoff value of 6.3 ng/mL. At this level, the sensitivity and specificity were 66% and 94%, respectively, with an area under the curve (AUC) of 0.803 (102).

Cystatin C (CysC) is a potent lysosomal cysteine protease inhibitor that plays a critical role in human vascular pathology by controlling tissue proteases and serving as a marker of renal function (105). A meta-analysis has shown that patients with IS exhibit significantly higher serum cystatin C concentrations compared to control groups (106). Zuo et al. found a U-shaped correlation between CysC levels and PSCI (107). A clinical study has also found that elevated serum CysC levels in IS patients were associated with a reduced risk of cognitive impairment at 3 months. This suggests that CysC may act as a protective factor against PSCI. However, it is important to note that this protective effect was only observed in patients with normal renal function and was not established in those with abnormal renal function. CysC may protect cognitive function by inhibiting oxidative stress in the post-IS period (108). Additionally, some studies have suggested that elevated serum CysC levels in the acute phase of IS could be an independent risk factor for PSCI. It is believed that CysC may contribute to cognitive dysfunction by promoting stenosis of the major arteries and increasing the risk of cerebral hemorrhage (109–111).

Fibrinogen, also known as coagulation factor I, plays a significant role in the development and progression of cardiovascular diseases (112). Elevated fibrinogen levels have been observed in patients with IS (113). In a study by Liu et al., blood fibrinogen levels were found to be higher in patients with PSCI compared to the non-PSCI group, and plasma fibrinogen levels were negatively correlated with Mini-Mental State Examination (MMSE) scores at 3 months (114). Additionally, a study by Roseborough et al. suggested that fibrinogen might be transmitted through NLRP3 signaling via an inflammatory response, which damages the blood-brain barrier (BBB) and contributes to cognitive dysfunction (115). Fibrinogen has also been shown to activate microglia, induce dendritic loss, and promote neuroinflammation, leading to synaptic deficits and cognitive decline (116).

Matrix metalloproteinases (MMPs) are a family of zinc-dependent endopeptidases, with MMP-9 being a prominent member of the gelatinase subclass (117). Some researchers have found that elevated serum MMP-9 levels are associated with impaired cognition after IS at three months (118). Serum MMP-9 levels have been shown to be negatively correlated with MoCA scores and cognitive functioning in patients with PSCI (119, 120). Furthermore, researchers have found that adding rheumatoid factor, MMP-9, and total homocysteine to conventional prediction models enhances the ability to predict PSCI (121). In contrast, the use of MMP inhibitors has been shown to improve the integrity of the blood-brain barrier (122). In an animal study by Lee et al., treadmill exercise before ischemia inhibited the activation of MMP-9, which improved short-term memory in rats (123). The precise mechanism by which MMP-9 contributes to PSCI remains unclear, and further research is needed to explore its potential as a therapeutic target for post-PSCI cognitive decline.

## 5 Discussions

As acknowledged, the pathogenesis of PSCI is complex and remains largely unknown. Current animal and clinical studies have found that PSCI is associated with inflammation (124), immunity (125), oxidative stress (126), intestinal flora (127), infections (128), metabolic syndrome (129), genetics (130), nutrition (131) and blood-brain barrier disruption (132). Many pathways, proteins, and molecules are involved, all of which affect the metabolism of sugars, proteins, and lipids inducing cognitive dysfunction. Inflammation is the most researched and well-studied mechanism (124).

In the early stages of IS, the interruption of oxygen and nutrient transport in the blood results in cell death and stress. Macrophages and other immune cells secrete damage-associated molecular patterns (DAMPs), which activate both local and peripheral immune responses, prompting the expression of a large number of inflammatory factors. Excessive inflammatory factors can enter the site of injury via the damaged blood-brain barrier (BBB), where they activate microglia, which can lead to PSCI by affecting signaling pathways such as TLR, JAK-STAT, NF-κB, and the purinergic P2 receptor family (133). The activation of these signaling pathways is linked to glucose, protein, and lipid metabolism (134–136). For instance, TLR4 activation increases glycolysis and the tricarboxylic acid (TCA) cycle in macrophages, while TLR8 signaling inhibits glucose metabolism in CD4+ Tregs by downregulating mTOR signaling (137, 138). Lipid rafts, cholesterol, fatty acid synthesis, and CD36-mediated binding of exogenous fatty acids contribute to MYD88 palmitoylation, which promotes TOLL-mediated inflammatory responses (139–141).

Moreover, stroke survivors with cognitive impairment exhibit increased inflammation, with C-reactive protein, IL-6, and TNF-α serving as predictors of PSCI (69, 142). Anti-inflammatory interventions have been shown to improve cognitive function in animal models, and therapies such as complement inhibition and fingolimod hold promise for reducing PSCI (124). Dietary restriction has also been found to improve cognitive dysfunction due to inadequate cerebral perfusion in IS patients by inhibiting inflammatory activation. Additionally, plant-based dietary patterns, including polyphenol-rich foods, can improve cognitive function by reducing oxidative stress and neuroinflammation while enhancing neurogenesis, synaptic plasticity, and neuronal survival (143). The role of inflammation and changes in substance metabolism in the prediction and diagnosis of PSCI should be further explored in future research.

The human APOE gene encodes apolipoprotein E (APOE), a ubiquitous lipid-transporting protein responsible for lipid metabolism in the bloodstream, as well as the transport of cholesterol and triglycerides. APOE exists in three common alleles: ϵ4, ϵ3, and ϵ2 (144). Clinical studies have found that elevated APOE4 gene expression increases the risk of IS, with the APOE-ϵ4 haplogroup being associated with dementia (130). Animal studies have shown that mice expressing the human APOE4 gene exhibit reduced expression of genes involved in oxidative phosphorylation in brain tissue, decreased pyruvate entry into the TCA cycle, and increased lactate production, resulting in reduced energy supply to the brain (145). Moreover, APOE4 has been found to inhibit fatty acid β-oxidation in astrocytes, disrupting brain fatty acid metabolism and bioenergetic homeostasis. These alterations have been associated with PSCI (146). These were associated with PSCI. Additionally, other studies have suggested that APOE4 can contribute to cognitive decline through mechanisms such as promoting hippocampal sclerosis (147), atherosclerosis (148, 149), and blood-brain barrier disruption (150).

The study finds that epigenetics is also associated with PSCI. Specifically, DNA methylation increases the risk of IS and is linked to a poor prognosis for IS patients (151, 152). Hypermethylation of the RIN3 gene in patients with PSCI negatively impacts cognitive function by promoting the accumulation of amyloid β-protein and tau protein phosphorylation (153).

Additionally, activation of the RING3 gene can regulate the promoters of genes through E2F transcription factors, which in turn affect the transcription and translation of proteins, potentially influencing protein metabolism (154). The study also discovered that DNA methylation levels were associated with both glucose metabolism and lipid metabolism (155, 156). However, it remains unclear whether DNA methylation is involved in PSCI through the regulation of substance metabolism, as no studies have yet reported this. Further research is needed to explore this potential mechanism in the future.

RNA is also associated with PSCI. MiR-126, a non-coding small RNA molecule, significantly reduces serum miR-126 expression after IS and may be involved in cognitive dysfunction by regulating angiogenesis, white matter (WM) remodeling, innate immune response, and inflammation (157). Meng et al. found that LncRNA MALAT1 improves cerebral ischemia-reperfusion injury and cognitive dysfunction by regulating the miR-142-3p/SIRT1 axis (158). However, whether RNA contributes to PSCI through the regulation of disruptions in glucose, proteins, and lipids has not been reported in any study.

In this paper, we systematically review the association between metabolic disorders and cognitive dysfunction following IS, with a particular focus on the changes in glucose, lipid, and protein metabolism and their associated biomarkers. Existing studies have demonstrated that metabolic disorders have complex and diverse mechanisms of action in PSCI. After IS, the disruption of blood flow to brain tissues obstructs the delivery of oxygen and glucose, causing glucose metabolism to shift to the anaerobic pathway. This shift activates the pentose phosphate pathway (PPP), leading to dysregulation of homeostasis in the intracerebral environment and negatively impacting cognitive function. Changes in lipid metabolism following IS are also significant. Under normal conditions, fatty acids are metabolized through both synthesis and β-oxidation. However, following IS, a reduction in ATP and acetyl coenzyme A production, inhibition of the tricarboxylic acid cycle, and activation of the hypothalamic-pituitary-adrenal (HPA) axis further inhibit fatty acid synthesis. In contrast, β-oxidation of fatty acids is enhanced after IS, providing substantial energy that aids in the functional recovery of brain tissue. Nevertheless, the decrease in total fatty acids and the overactivation of fatty acid β-oxidation can lead to oxidative stress, exacerbating brain tissue injury and contributing to PSCI. Therefore, the dual effects of lipid metabolic pathways warrant further investigation. Protein metabolism maintains homeostasis in the body primarily through degradation and synthesis pathways. After IS, endoplasmic reticulum (ER) stress in brain tissue rapidly induces protein misfolding via the unfolded protein response (UPR), which enhances protein degradation. Additionally, the hypoactivity of eIF2α and TORC1 after stroke inhibits protein synthesis and further promotes protein degradation. This metabolic disturbance ultimately leads to an imbalance between protein degradation and synthesis, contributing to cognitive dysfunction.

Future studies should incorporate advanced metabolomics and multi-omics techniques and interpretations from different perspectives to reveal the relationship between changes in metabolic pathways and cognitive dysfunction after IS. In addition, in future research on the pathogenic mechanisms and treatments for PSCI, it is important to consider not only the effects of the IS disease itself and medical treatment on cognitive function but also the role of underlying genetic factors in the development of cognitive impairment.

## 6 Conclusions

This article reviews the changes in glucose, lipid, and protein metabolism, along with their associated biomarkers, following IS, highlighting the significant role of metabolic disturbances in PSCI. It was found that these metabolic disturbances not only affect the energy supply but also impair cognitive function through complex mechanisms.

### 6.1 Limitations

Studies have shown that metabolites and metabolic pathways are influenced by various factors, including diet, medications, underlying diseases, and the time of onset (159). However, current research often does not account for these confounding factors, which may impact the findings. Future studies should focus on exploring the specific mechanisms of metabolic pathways and biomarkers while controlling for these variables.
